# Advancements in the Diagnosis and Treatment of Hypertrophic Cardiomyopathy: A Comprehensive Review

**DOI:** 10.3390/jcdd11090290

**Published:** 2024-09-18

**Authors:** Randeep Gill, Arsalan Siddiqui, Brianna Yee, Michael V. DiCaro, Nazanin Houshmand, Tahir Tak

**Affiliations:** 1 Department of Internal Medicine, Kirk Kerkorian School of Medicine at UNLV, Las Vegas, NV 89102, USA; randeep.gill@unlv.edu (R.G.); arsalan.siddiqui@unlv.edu (A.S.); brianna.yee@unlv.edu (B.Y.); michael.dicaro@unlv.edu (M.V.D.); nazanin.houshmand@unlv.edu (N.H.); 2 VA Southern Nevada Healthcare System, 6900 N. Pecos Road, North Las Vegas, NV 89086, USA

**Keywords:** alcohol septal ablation, heart failure, hypertrophic obstructive cardiomyopathy, mavacamten, mitral valve regurgitation, septal myectomy, systolic anterior motion

## Abstract

Hypertrophic cardiomyopathy (HCM) is characterized by excessive growth of myocardial tissue, most commonly due to genetic mutations in sarcomere proteins. This can lead to complications such as heart failure, mitral regurgitation, syncope, arrhythmias, sudden cardiac death, and myocardial ischemia. While we have come a long way in our understanding of the pathophysiology, genetics, and epidemiology of HCM, the past 10 years have seen significant advancements in diagnosis and treatment. As the body of evidence on hypertrophic cardiomyopathy continues to grow, a comprehensive review of the current literature is an invaluable resource in organizing this knowledge. By doing so, the vast progress that has been made thus far will be widely available to all experts in the field. This review provides a comprehensive analysis of the scientific literature, exploring both well-established and cutting-edge diagnostic and therapeutic options. It also presents a unique perspective by incorporating topics such as exercise testing, genetic testing, radiofrequency ablation, risk stratification, and symptomatic management in non-obstructive HCM. Lastly, this review highlights areas where current and future research is at the forefront of innovation in hypertrophic cardiomyopathy.

## 1. Introduction

Hypertrophic cardiomyopathy (HCM) was first described by Braunwald et al. in 1964 as “idiopathic hypertrophic subaortic stenosis” [1]. Its prevalence has been estimated to be roughly 1:500 worldwide [2,3]. Over the past 60 years, much has been discovered about the pathophysiology and genetic basis of this disease. HCM is characterized by hypertrophied myocardial tissue, most commonly due to genetic mutations in sarcomere proteins [4]. This can lead to a thickened left ventricle and interventricular septum, which, along with systolic anterior motion (SAM) of the mitral valve, can block the left ventricular outflow tract (LVOT) and cause obstruction [4]. This is referred to as hypertrophic obstructive cardiomyopathy (HOCM) and is responsible for the symptoms, disease progression, and mortality associated with HCM [5,6]. The past decade has seen exponential growth in the approach to the diagnosis and management of HCM, and this review aims to comprehensively analyze the current literature, with a look ahead to future research considerations.

## 2. Methods

We conducted a literature review using PubMed, MEDLINE, and Google Scholar databases. Keywords for this search included “hypertrophic cardiomyopathy”, “hypertrophic obstructive cardiomyopathy”, “HCM”, “genetics”, “diagnosis”, “treatment”, and “management”. We filtered search results to English language only, and the date range was from 2014 to 2024. Article types included observational studies, retrospective studies, epidemiological studies, randomized controlled trials, systematic reviews, meta-analyses, and textbook chapters. For updated current practice guidelines and societal recommendations on management, we referenced the “2024 AHA/ACC/AMSSM/HRS/PACES/SCMR Guideline for the Management of Hypertrophic Cardiomyopathy”.

## 3. Diagnosis

Recent advancements in imaging modalities and genetic testing have led to the development of new, comprehensive strategies for the diagnosis and monitoring of patients with HCM. Most patients initially present to their primary care physician or cardiologist due to a new onset of symptoms, known family history, or incidental findings on an electrocardiogram (EKG). A thorough personal and family history is critical in obtaining an accurate diagnosis and assessing disease severity. From there, imaging studies, ambulatory monitoring, and genetic testing may help to provide definitive diagnostic information.

### 3.1. History and Physical Examination

Initial discussions with patients should focus on the presence of symptoms, such as chest pain, exertional dyspnea, palpitations, and syncope. Identifying a trigger for these symptoms is also important, as symptoms tend to occur more frequently upon exertion. A thorough family history should be obtained, inquiring for a history of sudden cardiac death up to three generations. Often, patients with HCM may have cardiac auscultation findings including a crescendo–decrescendo murmur and a fourth heart sound.

### 3.2. Electrocardiogram

**Figure 1:**
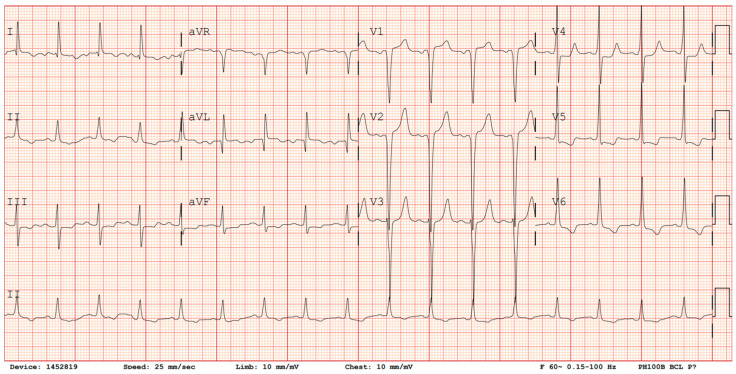
EKG demonstrating evidence of Q waves in V1–V2, deep S wave in lead V3, tall R waves in leads V4–V6, ST depression in V4–V6, and T-wave inversions in leads V5–V6.

A baseline EKG should be performed to aid in diagnosis and to assist in long-term monitoring. Diagnostic screening with EKG is advantageous as it is a simple, non-invasive, cost-effective tool that is readily available throughout a variety of practice settings. However, a disadvantage of the EKG as a diagnostic tool for HCM is that 4–6% of adult HCM patients will have a normal EKG, while many others will only display non-specific changes [7]. Electrical abnormalities seen on EKG in patients with HCM may include pathologic Q waves, deep S waves in leads V1–V3, high R waves in V4–V6, and T-wave inversions in V4–V6 (Figure 1). ST elevation may also be present in the anterior leads. While these findings may provide clues, often patients with HCM will only have mild, non-specific ST changes or T-wave abnormalities. At the time of initial diagnosis, ambulatory EKG monitoring via Holter monitor or wireless patch is usually indicated to evaluate for ventricular tachycardia (VT) and guide management on implantable cardioverter–defibrillator (ICD) placement [8,9]. Moreover, 12-lead EKG may be performed annually for monitoring asymptomatic changes in conduction, and follow-up ambulatory Holter monitoring may occur every 1–2 years in patients with no previous evidence of VT [9]. Individuals with positive gene mutations, but without expression of HCM phenotype, should undergo regular EKG monitoring every 5 years, or sooner in children and adolescents or if there is a change in clinical status [9].

### 3.3. Echocardiogram

Echocardiography is the most established imaging technique for the diagnosis of HCM and LVOT obstruction. Every initial visit should include a transthoracic echocardiogram (TTE) [9]. The immense value of echocardiography in the diagnosis of HCM lies in the ability to visualize septal thickening and left ventricular outflow tract obstruction, and it is also used to guide management for possible surgical intervention. Maximum left ventricular (LV) thickness of 15 mm or greater is usually diagnostic; in some instances, LV thickness of 13 mm or greater may be diagnostic as well, depending on symptoms and family history [9]. Specifically, LV thickening should be present in the absence of other cardiac, metabolic, rheumatologic, or other systemic processes. An echocardiogram allows for clear visualization of LVOT obstruction through several key parameters including systolic anterior motion (SAM) of the mitral valve, mechanical impedance, LVOT gradients, and visualization of mid-cavity muscular hypertrophy. One disadvantage of utilizing TTE for the diagnosis of HCM is in patients with dynamic LVOT obstruction that is only present during exercise, as this would not be diagnosed with standard echocardiography conducted at rest. If the LVOT gradient is minimal at rest but clinical suspicion for HOCM is still high, provocative testing with exercise or a pharmacologic echocardiogram can be performed [9]. TTE should be performed every 1 to 2 years thereafter to assess the progression of the disease.

### 3.4. Magnetic Resonance Imaging

Another valuable imaging modality in the diagnosis of HCM is cardiac magnetic resonance (CMR) imaging. Utilization of CMR is especially advantageous when echocardiography is equivocal, or additional information for invasive management is needed. Echocardiography and CMR both provide an assessment of LV thickness and mass, LVOT gradient, and ejection fraction. CMR can be further utilized to examine specific imaging features of the heart in patients with HCM, including increased right and left ventricular wall thickness, localized myocardial hypertrophy, apical aneurysm, myocardial fibrosis, papillary muscle hypertrophy or displacement, and left atrial fibrosis [10]. Given its higher resolution, contrast, and 3D mapping, it allows for a comprehensive evaluation of the myocardial tissue characteristics, morphology, and flow. Furthermore, the results are not operator-dependent, nor limited to acoustic windows that may otherwise limit the diagnostic accuracy as in echocardiography. Many CMR techniques are utilized to diagnose the phenotype, risk stratification, and determine treatment options in HCM. One such approach, called steady-state free precession, creates real-time, precise CMR images that allow an accurate measurement of localized wall thickness [11]. Another common technique is late gadolinium enhancement, which can identify extracellular abnormalities in the myocardium suggestive of fibrosis and is an independent predictor of adverse outcomes such as sudden cardiac death and heart failure [12,13,14]. In addition, CMR can characterize specific anatomic features in the mitral valve and papillary muscle, as well as hypertrophy distribution, which help determine the ideal therapeutic management for HCM patients, including for alcohol septal ablation and surgical myectomy [15,16]. The disadvantages of utilizing CMR as a diagnostic tool are that this modality is more costly and is not currently widely available.

### 3.5. Exercise Testing

Exercise testing is another diagnostic tool that can be utilized in the diagnosis of hypertrophic cardiomyopathy. As noted above, the downstream effects of HOCM can arise as a result of LVOT obstruction [5,6]. However, patients who undergo a standard diagnostic workup at rest may not be properly diagnosed if they only experience a dynamic left ventricular outflow tract obstruction during exercise. As such, exercise testing with stress echocardiography can be performed to evaluate the dynamic LVOT obstruction that is seen during exercise [9]. Cardiopulmonary exercise testing (CPET) is another form of exercise testing in which patients are assessed for certain variables including peak oxygen consumption, functional capacity, heart rate, and LVOT gradient. CPET is indicated to determine functional capacity in patients with non-obstructive HCM and advanced heart failure, pediatric and adult HCM patients during initial evaluation, HOCM patients, and as a screening every 2–3 years to evaluate for changes in functional capacity [9]. This evaluation of functional capacity can guide clinicians in the decision-making process for the treatment of HCM, which is discussed in detail below. Lastly, exercise stress testing can also be utilized to identify malignant arrhythmias that occur upon exertion, thus allowing these patients to further undergo risk stratification for the prevention of sudden cardiac death. While exercise testing can be beneficial in the diagnosis and management of HCM, a major disadvantage exists in that patients who are not able to perform strenuous exercise cannot be adequately screened with this modality.

### 3.6. Genetic Testing

Hypertrophic cardiomyopathy may be difficult to distinguish from other causes of left ventricular hypertrophy. Particularly, HCM must be distinct from hypertrophy due to long-standing hypertension, valvular heart disease, or ischemic heart disease. In this way, genetic testing can be beneficial in supporting the diagnosis of HCM. Genetic variants of sarcomere proteins that lead to disease have historically been used for diagnosis, though the inclusion of non-sarcomere abnormalities has also been debated, including in glycogen storage diseases or amyloidosis [17]. There is a clear relation to genetics in the prevalence of HCM. Specifically, more than 1000 genetic variations have been described in key genes such as ACTC, MYBPC3, MYH7, MYL2/3, TNNT2, TNNI3, TNNT2, and TPM1 [18,19,20]. Other associated genes are listed in Table 1 [4,21,22,23,24,25,26,27,28,29,30,31,32,33]. Naturally, different gene mutations may manifest as more severe forms of HCM, either through increased penetrance of the disease or earlier onset in life [18]. For example, mutations that encode for the myosin-binding protein C of the sarcomere were found to have reduced penetrance, as well as later disease onset [18]. Disease penetrance also varies within families, often with the most severe form of disease present in the proband. One disadvantage of genetic testing is that, although familial, the presence of a disease-causing genetic variant does not necessarily imply HCM diagnosis. Some individuals display incomplete disease expression or may be clinically unaffected altogether [20,34]. Subsequently, it is important to note that genetic testing does not have a known role in assessing the risk of sudden cardiac death. Individuals who are positive for pathogenic variants are still recommended to undergo surveillance with EKG, echocardiogram, and clinical assessment regularly [9]. Despite the variation in penetrance, recommendations still exist for genetic testing in an index patient with clinical suspicion of HCM, as well as for first-degree relatives of those with HCM [9]. Other indications for genetic testing include unexplained cardiac hypertrophy or to determine the familial inheritance for patients with diagnosed HCM.

## 4. Treatment

Treatment is not always indicated for patients diagnosed with HCM alone, and it is often reserved for individuals who display signs and symptoms of obstruction, as seen in HOCM. The goal of treatment is often to reduce LVOT obstruction by evaluating the LVOT gradient, as that is associated with worsening symptoms and mortality, though treatments may also provide dual benefit in decreasing arrhythmia episodes [5,6]. A summary of outlined treatment options is found in Table 2.

### 4.1. Beta-Blockers

For over 50 years, non-vasodilating beta-blockers have continued to be the mainstay first-line therapy for symptomatic patients with HOCM [9,35]. This class has been shown to significantly decrease the resting and provoked LVOT gradients in symptomatic patients with HOCM [35,36,37,38]. They have also been shown to decrease the degree of mitral regurgitation, increase left ventricular end-diastolic volume, and increase stroke volume in symptomatic patients [39]. These hemodynamic effects contribute to significantly improving exercise capacity [35] and the New York Heart Association (NYHA) functional class in these patients [36,38]. Beta blockade can also be initiated in patients with symptoms of angina or heart failure, even without evidence of HOCM. Beta-blockers have been shown to be very well tolerated, with only minimal and minor side effects [36]. Some studies have also suggested that beta-blockers have an association with mortality benefits and in decreasing the risk of sudden cardiac death (SCD), especially in younger patients [40,41,42]. Despite the clinical benefits, many patients continue to have symptoms even with increasing doses of beta-blocker therapy. The variability in treatment response across the population may arise due to genetic polymorphisms in beta-adrenergic receptors. One study analyzing this phenomenon demonstrated that beta-blockade significantly decreased NT-proBNP levels in most treatment groups; however, in glycine-389 homozygotes, this decrease was not statistically significant [37].

### 4.2. Calcium Channel Blockers

If patients continue to have symptoms despite beta-blocker treatment, it is recommended to supplement with a non-dihydropyridine calcium channel blocker, or switch to this class of medication if beta-blockers are not well tolerated [9]. Treatment with both classes of drugs should be used with caution, and close monitoring may be needed to evaluate for significant bradycardia and/or high-grade atrioventricular block [9]. Treatment of symptomatic HOCM with verapamil dates back to the late 1970s, with initial reports showing improvements in LVOT gradient, SAM, LV hemodynamic measurements, and LV muscle mass [43,44]. Further studies have shown that calcium channel blockers significantly reduce both resting and provoked LVOT gradients [45]. This contributes to an overall improvement in exercise capacity and NYHA functional class [46,47]. While calcium channel blockers are generally well tolerated, verapamil has been shown to have a slightly less favorable side effect profile compared to diltiazem [47,48]. Caution should be taken when initiating verapamil or diltiazem, particularly in severe LVOT obstruction and low systemic blood pressure, as a slight decrease in systemic blood pressure may paradoxically worsen outflow obstruction [9]. Dihydropyridine calcium channel blockers should be avoided in HOCM for the same reason [9].

### 4.3. Myosin Inhibitors

The newest major class of medications approved for the treatment of hypertrophic cardiomyopathy is cardiac myosin inhibitors. These medications are recommended to be added on for patients with persistent symptoms that failed monotherapy with either beta-blockers or calcium channel blockers [9]. Given the pathophysiology of HOCM involves hypertrophied myocardial tissue, pharmacological agents aimed at targeting this underlying process have been an area of significant interest over the past few years [4,49,50]. The PIONEER-HCM study was a landmark trial that introduced the allosteric myosin inhibitor mavacamten as a novel agent and demonstrated its effectiveness in decreasing post-exercise LVOT gradient in symptomatic HOCM patients [51]. Subsequent studies have shown that mavacamten can decrease resting and Valsalva LVOT peak gradients, resolve SAM, reduce symptoms and NYHA functional class, decrease NT-proBNP and cardiac troponin I, and increase peak oxygen uptake (pVO2) [52,53,54,55,56,57,58,59]. It has also demonstrated an improvement in cardiopulmonary exercise testing (CPET) measures such as METs, peak exercise time, and peak circulatory power, as well as non-peak CPET measures [60]. Mavacamten has also been shown to reduce the need to progress to more advanced therapy such as septal reduction therapy (SRT) [55,59,61]. Mavacamten has been found to have no statistically significant increase in serious adverse events (SAEs) between treatment versus placebo groups [56,57,62]. When monitoring for longer-term adverse outcomes, mavacamten has a small risk of causing atrial fibrillation and a transient reduction in left ventricular ejection fraction (LVEF) <50% [58].

Aficamten (CK-274) is a cardiac myosin inhibitor that was recently introduced as a next-in-class treatment of symptomatic HOCM [63]. The REDWOOD-HCM study demonstrated aficamten’s efficacy, with symptomatic patients seeing improvement in resting LVOT gradient, Valsalva LVOT gradient, LVEF, NYHA functional class, and NT-proBNP levels [64]. The SEQUOIA-HCM trial further highlighted the improved pVO2 demonstrated in patients taking aficamten, as well as improvements in NYHA functional class, eligibility for SRT, Valsalva LVOT peak gradient, and exercise capacity at 12 and 24 weeks [65]. While there has been no statistically significant difference in major adverse events noted between research groups administered aficamten versus placebo, one study showed that 3.5% of patients on aficamten developed LVEF <50% compared to 0.7% of placebo patients [64,65].

Close monitoring is required with the initiation of cardiac myosin inhibitors, due to their effects on left ventricular function and possible iatrogenic heart failure. These effects are, however, reversible with cessation of the medication. Given that cardiac myosin inhibitors are a novel class of medications, long-term safety and efficacy are currently a gap in the literature and will be an important area for extensive future study.

### 4.4. Disopyramide

Patients with symptoms refractory to beta-blockers or calcium channel blockers may be eligible for adding disopyramide, a class 1A antiarrhythmic, to their regimen [9,66]. Of note, it is highly recommended to be used concomitantly with an atrioventricular nodal blocking agent, to blunt its properties of accelerating AV nodal conduction in the event of atrial fibrillation [9]. Disopyramide has been shown in symptomatic HOCM patients to decrease resting LVOT gradient and improve NYHA functional class [67,68,69]. During exercise testing, disopyramide was found to decrease resting heart rate, peak heart rate, chronotropic response by age, pVO2, and functional capacity, while improving quality of life measures and NYHA functional class [70]. Disopyramide has been shown to have a favorable safety profile. Patient monitoring data revealed mild fatigue, weakness, anticholinergic symptoms, and a roughly 19-millisecond prolongation of QTc interval, but no clinically significant arrhythmias [68,71,72]. This may be due to an overall protective effect against ventricular arrhythmias. Along with its properties as a class 1A antiarrhythmic, disopyramide inhibits Na^+^, K^+^, and Ca^2+^ currents, reduces the action potential duration, and inhibits ryanodine receptors [68]. Disopyramide has also been shown to be associated with a delayed need for invasive management [73]. Patients who do not respond well to disopyramide are more likely to undergo further invasive management. These patients were more likely to have had worse pretreatment hemodynamic measurements on echocardiograms [74]. Another study suggests that a more favorable pretreatment NYHA functional class is also associated with better outcomes on disopyramide [72]. As such, these factors should be considered when initiating a patient on advanced therapies after failing first-line agents.

### 4.5. Ranolazine

Another less commonly used treatment option for symptomatic HOCM patients is ranolazine. Ranolazine has been shown to reduce the severity of microvascular angina and the burden of arrhythmias in these patients, and is relatively safe and well tolerated [75]. One study in symptomatic adult HOCM patients also demonstrated an improvement in angina and heart failure symptoms [76]. On the other hand, another randomized control trial showed that ranolazine showed no statistical difference from placebo in peak VO2, NT-proBNP, E/E′ ratio, or quality of life measures [77]. In an in vitro study using human cardiomyocytes, ranolazine was shown to reduce isometric contractility force during simulated exercise [78]. This is theorized to provide relief of obstructive symptoms of HCM that arise upon exertion and therefore can be considered in patients who otherwise cannot tolerate high doses of standard first-line therapies.

### 4.6. Septal Myectomy

Invasive management with septal reduction therapy (SRT) is indicated in HCM patients if symptoms are refractory to first-line medications [9]. The mainstays of SRT include surgical (or septal) myectomy (SM) and alcohol septal ablation (ASA). These patients are recommended to undergo evaluation with a transesophageal echocardiogram (TEE) for procedural mapping and guidance on specific SRT. Alternatively, patients may require CMR evaluation if TEE is inconclusive, as noted above. During septal myectomy, patients undergo an open sternotomy with cardiopulmonary bypass, followed by an aortotomy to access the septum for resection [79]. SM has been shown to significantly reduce the resting and provoked LVOT gradient in symptomatic HCM patients [79,80,81,82,83,84,85,86,87,88,89,90]. In addition, patients who undergo SM see an overall improvement in the NYHA functional class [79,80,81,82,83,84,85,86,87,89,90]. This procedure has also been shown to decrease the degree of MR and septal thickness [80,81,82,86,88,90,91]. While relatively well tolerated, certain arrhythmias such as right bundle branch block, left bundle branch block, and complete heart block have been seen after SM [92]. A small group of patients developed pacemaker dependency after the procedure, and this complication was seen as an independent risk factor for long-term mortality in HOCM patients. SM has also been shown to reduce the risk of HOCM patients subsequently developing atrial fibrillation [88,93]. Post-SM patients had improved survival compared to non-operated patients with similar circumstances [79,85,94]. Remarkably, in HOCM patients at 10 years post-SM, there was similar survival compared to the general population [87].

### 4.7. Alcohol Septal Ablation

Another invasive SRT option for symptomatic HOCM patients who are refractory to first-line treatments is alcohol septal ablation (ASA). This procedure is used as an alternative to SM in patients who are deemed too high-risk or who do not wish to undergo surgery [9]. First introduced in 1995 by Dr. Ulrich Sigwart, this technique uses a catheter-based approach to ablate the region of hypertrophied septal tissue with direct alcohol injection, which suppresses myocardial function and decreases intra-cavity pressure gradients in HOCM [95]. More recently, patients have undergone real-time intraoperative TEE to guide the procedural technique. There have been extensive studies conducted showing the effect of ASA in decreasing the resting and provoked LVOT gradients in symptomatic HOCM patients [79,96,97,98,99,100,101,102,103,104,105,106,107,108,109,110]. Alcohol septal ablation has also been shown to improve the NYHA functional class in the post-treatment analysis [79,96,97,98,99,101,102,104,105,106,107,108,109,110]. The effects of ASA in decreasing septal thickness [96,98,102,103] and improving LV diastolic function [111] have likely contributed to its effect on increasing exercise tolerance [106] and decreasing levels of NT-proBNP [103,109]. Common conduction abnormalities that develop after undergoing ASA include an estimated 8–10% risk of developing a complete heart block requiring a permanent pacemaker [96,97,98,101,102,110,112,113,114,115,116,117,118], right bundle branch block [119], and atrial fibrillation [120]. Post-ASA, elderly patients were more likely to experience major adverse cardiovascular events (MACEs) and AV nodal block compared to younger patients [121,122,123,124]. ASA has approximately a 1% risk of post-procedure mortality within the first year [125]. Post-ASA survival after 5 years is approximately 89–96%, compared with 78–80% for non-invasive patients [79,99,106,113,114,126]. Just as in SM, long-term survival after ASA has been shown to be similar to that of the general population [98,113,116].

### 4.8. Radiofrequency Ablation

Radiofrequency ablation (RFA) is an alternative invasive, catheter-based septal reduction therapy, that can be utilized in patients who are not good candidates for SM and have cardiac anatomy that is not amenable to ASA [127]. RFA has been shown to reduce the resting and provoked LVOT gradient in symptomatic HOCM patients by producing septal hypokinesis and causing ineffective contraction in the myocardium that would otherwise lead to outflow obstruction [128,129,130,131,132,133,134,135,136,137]. Radiofrequency ablation has demonstrated significant improvement in the post-treatment NYHA functional class [128,129,130,131,133,135,136,137]. RFA also reduces the interventricular septal thickness [131,133,136,137]. In one study, patients treated with RFA had a decreased QRS amplitude in leads V1 and V2 on their follow-up EKG, also suggesting a reduction in septal thickness [138]. RFA additionally improved the overall duration of cardiopulmonary exercise testing [129,131,133,134,135]. This technique has not been shown to cause heart block requiring pacemaker placement [131,133] or malignant ventricular arrhythmias [136]. RFA has, however, been shown to have a slightly increased risk of causing a right bundle branch block [133].

### 4.9. Management of Non-Obstructive HCM

Patients with hypertrophic cardiomyopathy without significant LVOT gradient, also referred to as non-obstructive HCM, may still develop symptoms. These symptoms, including angina and dyspnea, are more pronounced with exertion and warrant symptomatic management. Similar to HOCM, the mainstay medical treatment for symptomatic non-obstructive HCM is with beta-blockers or calcium channel blockers. Beta-blockers have been shown to reduce the mean and maximal heart rate and decrease NT-proBNP levels [37]. This can contribute to improving diastolic function and decreasing the overall myocardial oxygen demand, thus providing symptomatic relief [9,35,37]. Non-dihydropyridine calcium channel blockers have also been shown to reduce symptoms and increase exercise capacity in HCM patients [48]. In addition, these medications decrease myocardial ischemia in patients with HCM, thus reducing angina experienced upon exertion [139]. However, beta-blockers or calcium channel blockers are not indicated in asymptomatic patients with non-obstructive HCM [9].

### 4.10. Implantable Cardioverter–Defibrillator

Given that HCM confers a substantial risk of sudden cardiac death (SCD), patients should be risk-stratified at initial evaluation and every 1–2 years for implantable cardioverter–defibrillator (ICD) placement. It should be noted that ICD placement is a preventative measure against SCD, and is not a definitive treatment option for HOCM. Variables associated with an increased risk of SCD include a personal or family history of cardiac arrest or sustained ventricular arrhythmia, a cardiac syncopal event within the previous 6 months, LV systolic dysfunction, significant LV wall thickness, or LV apical aneurysm [9]. In patients deemed high-risk for SCD, shared decision-making regarding ICD placement for primary prevention of SCD is recommended. In HCM patients with a previous cardiac arrest and/or sustained ventricular arrhythmia, secondary prevention with ICD placement is strongly recommended [9].

## 5. Future Directions

Given the extensive literature to date on the management of hypertrophic cardiomyopathy, there is a significant opportunity to expand on the current knowledge and advance future research endeavors. As the availability and utilization of genetic testing increases, the amount of information from which physicians can make management decisions will be extensive. As is evident in patients non-responsive to beta-blockers seemingly due to a genetic mutation, the genetic profiles of patients can aid in choosing appropriate medication regimens. Additionally, these genetic profiles may even allow physicians to initiate treatment before a diagnosis of HCM is formally made.

One topic area at the forefront of current and future research interest is artificial intelligence (AI). As the processing power and advanced algorithms of AI have developed into machine learning and deep learning networks, researchers have developed AI tools capable of diagnosing HCM via EKG interpretation [140]. Some such tools have shown impressive diagnostic accuracy and external validity [141,142]. As artificial intelligence models develop further, they will have a significant role in the diagnosis, treatment, and risk stratification of HCM in the near future.

Another area in which future study will be largely beneficial is in the long-term safety and efficacy profiles of newer medication classes, specifically the cardiac myosin inhibitors. As this class of medications has changed the landscape of current treatment, it will be important to compare head-to-head with the other pharmacological and procedural options as a viable long-term therapy. New medications that are currently being studied in animal and early human trials will be an area of extensive interest. Two such medications, ninerafaxstat and omecamtiv mecarbil, have already shown promising early results.

Innovations in SRT, including a transapical approach in SM and alternative access sites for ASA, present a continued opportunity to improve upon invasive techniques. Additionally, dosage and choice of alcohol agent in SM is another area for researchers to explore.

## 6. Conclusions

Hypertrophic cardiomyopathy has been an area of significant study over the past decade. As the body of evidence has grown for the diagnosis and treatment of HCM, physicians have multiple options for management, including newer modalities outlined in this review. HCM patients exist across a wide spectrum of demographics, comorbidities, presentations, and perspectives. Establishing a shared decision-making approach is necessary for practicing high-quality, patient-centered care. As such, we emphasize the importance of taking into account each patient’s unique circumstances to guide management. This also highlights the continued opportunity for future research into safety, efficacy, and innovative approaches to treatment. Given the strong foundation of the established literature, current and future researchers can build upon this knowledge to continue making further advancements for years to come.

## Figures and Tables

**Table 1:** Genes associated with HCM (alphabetical) [4,21,22,23,24,25,26,27,28,29,30,31,32,33].

Gene	Protein End Product
ACTC	α-cardiac actin
ACTN2	α-actinin2
ALPK3	α-kinase 3
MYOZ2	Myozenin2
CASQ2	Calsequestrin
CAV3	Caveolin-3
CSRP3	Muscle LIM protein
FHL1	Four-and-a-half LIM domains 1
FLNC	Filamin C
JPH2	Junctophilin 2
MYBPC3	Cardiac myosin-binding protein C
MYH6	α-myosin heavy chain
MYH7	β-myosin heavy chain
MYL2	Regulatory myosin light chain
MYL3	Essential myosin light chain
PLN	Phospholamban
TCAP	Telethonin
TNNT2	Cardiac troponin T
TNNI3	Cardiac troponin I
TPM1	α-tropomyosin
TRIM63	Muscle RING-finger protein-1
TTN	Titin
VCL	Vinculin

**Table 2:** Summary table of pharmacological and non-pharmacological treatment options for HOCM.

	Treatment	Definitive?	Method of Improvement
Pharmacological treatment	Beta-blockers	No	Decreases heart rate and LVOT gradient Improves mitral regurgitation Decreases frequency of arrhythmias
	Calcium channel blockers	No	Decreases heart rate and LVOT gradient Decreases SAM Decreases LV muscle mass
	Cardiac myosin inhibitors	No	Decreases LVOT gradient Decreases SAM
	Disopyramide	No	Decreases heart rate and LVOT gradient Decreases frequency of arrhythmias
	Ranolazine	No	Reduces microvascular angina Decreases frequency of arrhythmias
Non-pharmacological treatment	Septal myectomy	Yes	Surgical removal of obstructive tissue
	Alcohol septal ablation	Yes	Localized decrease in myocardial function → decreases intra-cavity pressure gradients
	Radiofrequency ablation	Yes	Localized decrease in myocardial function → decreases intra-cavity pressure gradients Decreases septal thickness
	Implantable cardioverter–defibrillator	No	Termination of ventricular tachycardia to avoid sudden cardiac death

## Data Availability

All the data reported are present on PubMed, MEDLINE, and Google Scholar web databases.
